# Platelet distribution width—a prognosis marker in patients with chronic heart failure

**DOI:** 10.1097/j.pbj.0000000000000277

**Published:** 2025-01-08

**Authors:** Ana Neves, Rita Gouveia, Sérgio Madureira, Catarina Elias, Pedro Ribeirinho-Soares, Marta Soares-Carreira, Joana Pereira, Jorge Almeida, Patrícia Lourenço

**Affiliations:** a Internal Medicine Department, Unidade Local de Saúde São João, Porto, Portugal; b Department of Medicine, Faculty of Medicine, Porto University, Porto, Portugal

**Keywords:** chronic heart failure, platelet distribution width, prognosis

## Abstract

**Background:**

Increased levels of platelet distribution width (PDW) can predict cardiac death and infarction recurrence in acute myocardial infarction. PDW appears to be a prognosis marker in acute heart failure (HF); however, its impact on chronic HF is still unknown. We investigated the impact of PDW on chronic HF.

**Methods:**

We retrospectively analyzed outpatients with chronic HF with left ventricular systolic dysfunction (LVSD) from January 2012 to May 2018. Patients with no data on PDW levels or with preserved or recovered ejection fraction were excluded. The primary end point was all-cause mortality. Multivariable Cox regression analysis was used to evaluate the association between PDW and mortality. A multivariate model was built adjusting for age, sex, comorbidities, brain-type natriuretic peptide, New York Heart Association (NYHA) class, evidence-based therapy, and severity of LVSD.

**Results:**

In our cohort of 766 patients, 65.7% were male, the mean age was 70 years, and 35.4% were in NYHA class I; 38.3% had diabetes mellitus, 51.4% had severe LVSD, and 3.9% had an inflammatory or autoimmune disease. The median (interquartile range) PDW was 13.5 (12.1–14.9) fL. During a median follow-up of 49 (30–79) months, 372 patients (48.6%) died. Patients with PDW ≥ 14.3 fL presented a multivariate-adjusted higher risk of all-cause death than those with lower values (hazard ratio: 1.32, 95% confidence interval [CI]: 1.05–1.64, *P* = .2).

**Conclusions:**

Patients with PDW ≥14.3 fL (upper tercile for PDW) presented a multivariate-adjusted 32% (95% CI: 5–64%) higher risk of all-cause death than those with lower values. PDW can help clinicians stratify patients with chronic HF; it is a practical, inexpensive, and widely available parameter.

## Abstract 2

Supplemental Digital Content is Available in the Text.

## Introduction

Chronic heart failure (HF) is a prevalent and growing problem in an increasingly old population worldwide.^1-3^ The overall 5-year mortality for those with HF is high (52.6%).^3^ An adequate risk stratification in patients with HF is required to estimate prognosis, define management strategies, and allocate resources.^3^

A laboratory evaluation that includes a complete blood count is recommended by international guidelines.^2,4^ The prognostic value of hemoglobin and hematocrit is firmly established for patients with HF.^5^ However, a complete blood count extends widely beyond the hemoglobin and hematocrit values, and it also portends information about many other parameters, such as platelet count and platelet distribution width (PDW). The PDW corresponds to a marker of platelet activation, measuring the variability in platelet size. Larger platelets are considered metabolically and enzymatically more reactive.^6^ Increased levels of PDW are associated with atherosclerosis, coronary artery disease (CAD), cerebrovascular disease, and systemic inflammation.^7-12^ All these are known to play key roles in the pathophysiology of HF.^2^ The association of PDW with HF severity and prognosis is widely unknown. Higher levels of PDW have been previously associated with an increased risk of cardiac death and infarction recurrence in patients presenting with acute myocardial infarction.^8,11^ For the first time, in 2020, Sato and colleagues reported that higher PDW values are predictors of worse outcomes in patients with acute HF^13^; in 2023, an inverse relationship between PDW and mortality was reported in an acute setting.^14^ However, the prognostic impact of PDW levels was never addressed in the chronic HF setting.

We aimed to investigate the prognostic impact of PDW in patients with chronic HF with left ventricular systolic dysfunction. We hypothesized that elevated PDW values would also predict more ominous outcomes in chronic HF.

## Materials and methods

We retrospectively investigated outpatients with chronic HF with systolic dysfunction followed between January 2012 and May 2018 in the Internal Medicine Department of Centro Hospitalar Universitário São João (CHUSJ). CHUSJ is a Portuguese tertiary care academic hospital that assists in an area of more than 320 thousand inhabitants. Patients followed in the HF clinic of the Internal Medicine Department are mainly patients with HF with reduced ejection fraction (EF ≤ 40%), and patients with HF with mildly reduced ejection fraction were also followed. The referral comes mainly from the internal medicine ward and primary care facilities; a smaller proportion of patients is referred from the emergency department and also from other specialties, including cardiology, for patients with multiple and complex comorbidities.

Adult patients (18 years or older) with HF and left ventricular systolic dysfunction observed in our HF clinic between the abovementioned time frame were eligible for study inclusion. HF diagnosis was made according to updated guidelines.^2,15,16^ Patients with ejection fraction between 40% and 49% were classified as having HF with mildly reduced ejection fraction and those with ejection fraction <40% as having HF with reduced ejection fraction (HFrEF).^2^ Within patients with HFrEF, ejection fraction <30% was considered as severe systolic dysfunction. Demographic data, comorbidities, clinical and laboratory parameters, and medication in use at the index visit were recorded. The index visit was considered the first patient evaluation since January 2012. Patients with HF with preserved or totally recovered ejection fraction and patients with no data on PDW levels on the first appointment were excluded.

B‐type natriuretic peptide (BNP) determination was made through an Abbott chemiluminescent microparticle immunoassay (two‐step immunoassay). Hemoglobin was obtained using an automated blood counter Sysmex XE‐5000. PDW was analyzed from anticoagulated blood (using K3-ethylenediaminetetraacetic acid) using an automated hematology analyzer, Sysmex XE (Sysmex Europe SE). This procedure was performed by experienced laboratory technicians at Centro Hospitalar Universitário de São João who were independent of this study. Serum creatinine was measured through conventional methods with an Olympus AU5400 automated clinical chemistry analyzer (Beckman‐Coulter). The glomerular filtration rate (GFR) was estimated according to the Modification Diet and Renal Disease (MDRD) formula.^17^

Arterial hypertension was defined as systolic blood pressure record ≥140 mmHg and/or diastolic blood pressure ≥90 mmHg in at least 2 separate measurements; the presence of diabetes mellitus (DM) was defined as either a known previous diagnosis, current prescription of hypoglycemic agents, fasting venous blood glucose above 126 mg/dL, random glucose >200 mg/dL, or glycosylated hemoglobin (HbA1c) ≥ 6.5%. CAD was defined by the presence of a history of acute myocardial infarction or important CAD confirmed by imaging methods. Cerebrovascular disease was considered when patients presented with a history of stroke or cerebral hemorrhage or in cases where a cerebral vascular lesion image was confirmed by an imaging method. Peripheral artery disease was considered in patients with a previously known and reported diagnosis, ankle-brachial index measurement <0.9, or a significant arterial narrowing due to atherosclerosis confirmed by imaging methods. Noncoronary artery disease included cerebrovascular disease and/or peripheral artery disease.

The end point of this study was all-cause mortality. Patients were followed from the first medical appointment in 2012 until January 2021. We determined the patients' vital status by consulting hospital registries and by telephone contact with the patients or their relatives. When no information was obtained, we consulted the patients' national registry (Registo Nacional de Utentes) platform. The causes of death are not discussed in this article.

The registry's protocol followed the ethical guidelines of the Declaration of Helsinki, and it was approved by the local ethics committee. Because of the retrospective nature of the study design, informed consent was not performed.

### Statistical analysis

Mean ± standard deviation was used for continuous variables with a normal distribution and median (interquartile range) for non-normally distributed continuous variables. Categorical variables are described as counts and proportions. The distribution of PDW was depicted in the form of a histogram. Patients were categorized according to initial PDW terciles: 1st group with PDW < 12.6 fL, 2nd group ≥12.6 and <14.3 fL, and 3rd group ≥14.3 fL. The Kaplan–Meier method was used to study the survival curves according to the PDW terciles. Since patients in the first and second groups had similar long-term survival, the PDW value of 14.3 fL (corresponding to 66.7th percentile of the distribution) was the chosen cutoff. Patients with PDW below and above the cutoff were compared: the chi-square test for categorical variables, the Student *t* test for normally distributed continuous variables, and the Mann–Whitney U test for continuous variables with non-normal distribution. Multivariable Cox regression analysis was used to assess the association between PDW and all-cause mortality, adjusting for potential confounders. PDW was analyzed both as a categorical variable (cutoff 14.3 fL) and as a continuous variable (per 1-fL increase in the PDW value). Three multivariate models were built, with successive models adjusting for increasing number of potential confounders. In model 1, adjustments were made considering age, sex, CAD, noncoronary atherosclerotic disease, DM, and inflammatory/autoimmune disease. Model 2 included variables in model 1 plus New York Heart Association (NYHA) class, left ventricular systolic dysfunction severity, chronic kidney disease, and B-type natriuretic peptide. Model 3 included the variables in model 2 and also beta-blocker (BB) use, renin–angiotensin system inhibitor (RASi) use, and mineralocorticoid receptor antagonist (MRA) use.

## Results

**Figure 1.**
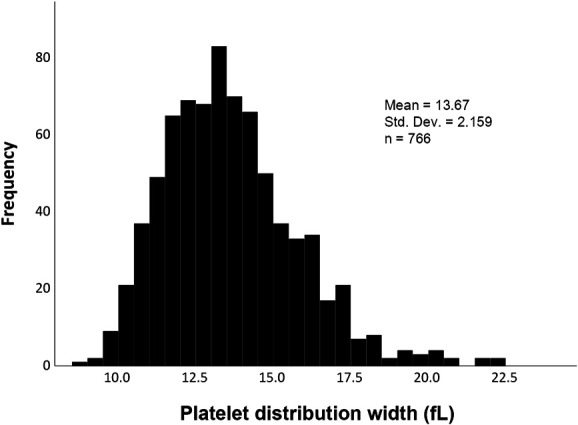
PDW distribution of the study cohort.

We studied 766 ambulatory patients with chronic HF with left ventricular ejection fraction <50%; 65.7% were male, the mean age was 70± 13 years, 35.4% were in NYHA class I, 44.9% were in NYHA class II, and the remaining were in higher classes. DM was present in 38.3%, 51.4% had severe left ventricular systolic dysfunction, and 3.9% had an inflammatory or autoimmune disease. At the end of the index appointment, 93.2% were on beta-blockers, 84.5% on RASis, and 29.5% on mineralocorticoid receptor antagonists. The median (interquartile range) BNP value was 281.9 pg/mL (119.1–637.9 pg/mL). Patient characteristics are shown in Table 1. The PDW distribution of the study cohort is shown in the form of a histogram in Figure 1.

**Table 1:** Patient characteristics and comparison between those with PDW < 14.3 fL and those with higher values.

Characteristics	All (n = 766)	PDW < 14.3 fL	PDW ≥ 14.3 fL	*P*
Age (y), mean (SD)	70 (13)	70 (13)	71 (13)	.50
Male sex, n (%)	503 (65.7)	319 (64.3)	184 (68.1)	.29
Arterial hypertension, n (%)	463 (60.4)	289 (58.3)	174 (64.4)	.10
Diabetes mellitus, n (%)	293 (38.3)	169 (34.1)	124 (45.9)	.001
Coronary artery disease, n (%)	354 (46.2)	228 (46.0)	126 (46.7)	.85
Noncoronary atherosclerotic disease, n (%)	185 (24.2)	128 (25.9)	57 (21.2)	.15
Severe LVSD, n (%)	394 (51.4)	246 (49.6)	148 (54.8)	.17
Inflammatory/autoimmune disease, n (%)	30 (3.9)	24 (4.4)	6 (2.2)	.08
Hemoglobin (g/dL), mean (SD)	13.2 (1.8)	13.1 (1.8)	13.3 (1.9)	.24
eGFR (mL/min/1.73 m^2^), median (IQR)	56 (40–75)	58 (42–78)	51 (36–69)	.001
BNP (pg/mL), median (IQR)	281.9 (119.1–637.9)	253.8 (107.1–560.9)	320.2 (159.3–718.4)	<.001
NYHA ≥ III, n (%)	151 (19.7)	94 (19.0)	57 (21.2)	.47
Beta-blockers, n (%)	714 (93.2)	460 (92.7)	254 (94.1)	.48
RASi, n (%)	647 (84.5)	424 (85.5)	223 (82.6)	.29
MRA, n (%)	226 (29.5)	153 (30.8)	73 (27.0)	.27
Follow-up (mo), median (IQR)	49 (30–79)	49 (31–78)	47 (28–80)	.45
Mortality, n (%)	372 (48.6)	223 (45.0)	149 (55.2)	.007

BNP, B-type natriuretic peptide; eGFR, estimated glomerular filtration rate; IQR, interquartile range; LVSD, left ventricular systolic dysfunction; MRA, mineralocorticoid receptor antagonist; NYHA, New York Heart Association; RASi, renin–angiotensin system inhibitor; SD, standard deviation.

**Figure 2.**
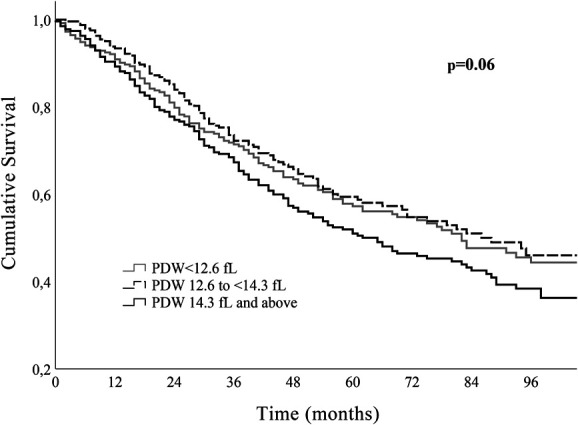
Survival curves according to PDW tertiles. Patients with PDL < 12.6 and between 12.6 and 14.3 have similar survival.

**Figure 3.**
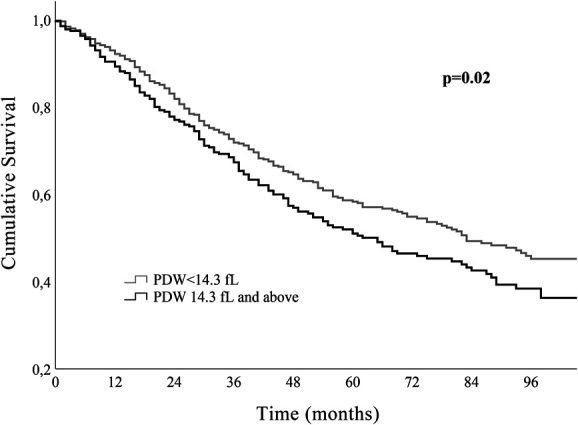
Survival curves according to PDW values: <14.3 fL and ≥14.3 fL. Patients in the last PDW tertile presented higher long-term mortality.

PDW presented a slightly skewed right-tailed distribution. The median PDW was 13.5 fL; percentile 33.3 corresponded to the value 12.6 fL and percentile 66.7 to 14.3 fL. Comparison between patients with PDW < 14.3 and those with PDW ≥ 14.3fL are also presented in Table 1. Patients with higher PDW values presented with significantly higher BNP values and lower glomerular filtration rates. Diabetes was also more prevalent among patients with PDW values above 14.3 fL (45.9% versus 34.1% in patients with lower PDW values). During a median follow-up of 49 (30–79) months, 372 patients (48.6%) died: 45.0% in those with PDW < 14.3 fL and 55.2% in those with higher basal PDW (*P* = .007). The comparison between the three terciles can be seen in Table 1 of the Supplemental Digital Content (http://links.lww.com/PBJ/A40). Kaplan–Meier survival curves according to the PDW terciles are shown in Figure 2. The group of patients with clear survival disadvantage corresponded to the patients in the last tercile, while patients in the first and second terciles had nondifferent survival curves. Figure 3 shows the survival curves of patients with PDW< and ≥14.3 fL, and those with PDW ≥ 14.3 fL presented a clear survival disadvantage in the long term.

Patients with PDW >14.3 dfL presented a crude hazard ratio (HR) of all-cause mortality of 1.27 (95% confidence interval [CI]: 1.03–1.56, *P* = .024). Table 2 presents the multivariate-adjusted association of higher PDW with all-cause mortality. In sequentially complex models adjusting for additional potential confounders, the independent association of higher PDW with increased risk of all-cause mortality sustained. Patients with PDW ≥ 14.3 fL presented a multivariate-adjusted HR of death of 1.32 (95% CI: 1.05–1.64, *P* = .2) independent of age, sex, CAD, noncoronary atherosclerotic disease, DM, inflammatory/autoimmune disease, NYHA class, LVSD severity, chronic kidney disease, BNP, and evidence-based therapy. When PDW was analyzed as a continuous variable, the increase in risk was of 5% per each 1-fL rise in PDW.

**Table 2:** Crude and multivariate associations of PDW ≥ 14.3 fL and PDW analyzed as a continuous variable.

	PDW ≥ 14.3 fL		PDW per 1-fL increase	
	HR (95% CI)	*P*	HR (95% CI)	*P*
Crude	1.27 (1.03–1.56)	.02	1.05 (1.00–1.10)	.04
Multivariate-adjusted model 1*	1.29 (1.04–1.59)	.02	1.05 (1.00–1.10)*	.03
Multivariate-adjusted model 2†	1.32 (1.06–1.64)	.02	1.05(1.00–1.10)†	.05
Multivariate-adjusted model 3‡	1.32 (1.05–1.64)	.02	1.05 (1.00–1.10)‡	.05

Models are increasingly complex.

* Adjustments considering age, sex, coronary artery disease, noncoronary atherosclerotic disease, diabetes mellitus, and the coexistence of an inflammatory/autoimmune condition.

† Adjustments for variables considered in model 1 + New York Heart Association class, severity of left ventricular systolic dysfunction, chronic kidney disease, hemoglobin, and B-type natriuretic peptide.

‡ Adjustments for variables in model 2 + beta-blocker use, renin–angiotensin system inhibitor use, and mineralocorticoid receptor antagonist use.

## Discussion

In our cohort of patients with chronic HF with systolic dysfunction, elevated PDW values were associated with all-cause mortality in the long term. Patients with PDW ≥ 14.3 fL had an independent higher risk of 32% (95% CI 5–64%) of all-cause death when compared with the remaining. The increase in risk was of 5% per each fL increase in PDW.

Platelets are produced by megakaryocytes in the bone marrow and are found in peripheral blood. Platelets are known to play a central role in homeostasis, but it is currently recognized that these small anucleate cellular fragments are highly complex and metabolically active cells. Platelets are also active players of inflammatory and innate immune responses. Activated platelets secrete various inflammatory mediators and gain the ability to adhere with neutrophils, monocytes, and even lymphocytes. The ability to interact with both the vascular endothelium and leucocytes is proof of the link between inflammation, thrombosis, and atherogenesis.^18-20^ These observations reinforce the importance of antiplatelet therapy in the prevention of cardiovascular events but open a wide window of possibilities in inflammatory conditions.^20^ Mean platelet volume and PDW are important indexes that reflect platelet production and, indirectly, may relate to platelet function and activity. Alterations in their volume and volume distribution are known to reflect a proinflammatory state and oxidative stress.^21,22^ PDW does not increase during simple platelet swelling, being a more specific marker of platelet activation.^23^

Diabetes, smoking habits, obesity, renal and atherosclerotic diseases, both coronary and non-coronary, are known to course with increased production and release of proinflammatory cytokines, with tissue hypoxia, and organ damage. Platelets are an active part of this inflammatory response, both triggering and enhancing it. This proinflammatory milieu courses with platelet activation, increased platelet turnover, and, therefore, increased platelet volume and platelet distribution width.^22,24,25^

Chronic HF can be considered a low-grade inflammatory condition, and therefore, we can suppose that platelet activation may play a role in disease progression.^26^ Elevated PDW values have already been associated with worse prognosis in acute patients presenting with acute HF in a 10-year prospective study in Fukushima Medical University Hospital.^13^

Numerous biomarkers have been studied and proposed for HF prognostication; still, an accurate risk stratification remains challenging, and no single marker has proven accurate enough. BNP is the most studied and widely used biomarker to manage and stratify patients with chronic HF; however, it has important limitations concerning interpretation in patients with atrial fibrillation, obesity, and renal dysfunction.^27^ More recently proposed biomarkers such as soluble suppression of tumorigenicity 2 (sST2),^28^ growth differentiation factor 15 (GDF-15),^29^ and galectin-3^30^ have been proven to portend prognostic information in HF but are still not widely available. New biomarkers could improve the understanding of HF physiopathology and make individualized therapies improving patients' survival and quality of life.

To the best of our knowledge, this is the first time PDW was evaluated as a prognostic marker in patients with chronic HF and we report an independent association of elevated PDW with higher mortality risk. Its prognostic value was independent of age and sex, concomitant comorbidities, symptoms, severity of LVSD, evidence-based therapy, and BNP. The only reports suggesting an association between PDW and outcomes in HF were in the acute HF setting. Sato^13^ found a U-shaped association of PDW values and mortality; however, patients in the third tercile of PDW distribution showed a clear survival disadvantage when compared with the remaining groups.^13^ In this study, P33.3 and P66.7 were 15.9 and 16.9, respectively, clearly higher than the PDW values in our patient population; this difference is probably explained by the fact that patients in Sato's study were hospitalized because of acute HF decompensation, therefore, in a so-called flare of their chronic low-grade inflammatory condition. It is interesting to note that in the referred study in acute HF, the mean ejection fraction was more than 50% and, therefore, it was a population of patients mainly with HF with preserved ejection fraction; the fact that the reported all-cause mortality was 22.3% during a median follow-up of approximately three and a half years is somehow in contrast to the reported elevated mortality in HF in general, particularly in acute HF.^3^ Our cohort represents a more uniform group of not-preserved chronic HF patients, and the mortality of 48% in a median 4-year follow-up is more in accordance with what is the described HF mortality. The reported finding of an inverse association of PDW with adverse outcomes^14^ is contrary to existing literature and counterintuitive based on its recognized association with inflammatory activation.^18-20^

Several limitations to our study should be noted. First, it is a retrospective single-center study that precludes its generalization. Second, patients included in our cohort were not under drugs currently recognized as clear prognostic-modifying in HF with reduced ejection fraction. Third, adjustments were made considering the coexistence of autoimmune or inflammatory conditions; however, C-reactive protein was not routinely measured and could not be accounted for; the coexistence of active cancer was also not accounted for. Finally, the retrospective nature precludes the establishment of a causal association between PDW values and mortality.

Despite these limitations, this is the first study reporting the association of elevated PDW with all-cause mortality in patients with chronic HF. This association does not mean causality, and as with other inflammatory markers in HF, we cannot conclude whether they are implicated in HF progression or are simply markers of disease severity. Nonetheless, elevated PDW showed to be a mortality predictor irrespective of age, comorbidities, and even BNP; therefore, whether casually implicated or not in this ominous outcome, it may help clinicians identify higher-risk groups among chronic HF populations. This is even more important if we consider that PDW is a common and directly available parameter that is almost universally ignored by physicians treating patients with HF. More ambitious, however possible, future implications could be looking into platelets and platelet activation as targets for new or more tailored therapeutic approaches in HF. Additional studies would be necessary to support our results, but at least the potential for a more accurate risk stratification should be clarified because it is a broadly available parameter in clinical practice.

## Conclusions

PDW is an independent mortality predictor in patients with chronic HF. Patients with PDW ≥ 14.3 fL (upper tercile for PDW) presented a multivariate-adjusted 32% (95% CI: 5–64%) higher risk of all-cause death than those with lower values. PDW can help clinicians to identify high-risk patients with chronic HF, being a practical, inexpensive, and widely available parameter.

## References

[1] Brouwers FP de Boer RA van der Harst P . Incidence and epidemiology of new onset heart failure with preserved vs. reduced ejection fraction in a community-based cohort: 11-year follow-up of PREVEND. Eur Heart J. 2013;34:1424–31. 23470495 10.1093/eurheartj/eht066

[2] McDonagh TA Metra M Adamo M , ESC Scientific Document Group. 2021 ESC Guidelines for the diagnosis and treatment of acute and chronic heart failure. Eur Heart J. 2021;42:3599–726. 34447992 10.1093/eurheartj/ehab368

[3] Tsao CW Aday AW Almarzooq ZI , American Heart Association Council on Epidemiology and Prevention Statistics Committee and Stroke Statistics Subcommittee. Heart disease and stroke statistics-2023 update: a report from the American Heart Association. Circulation. 2023;147:e93–621. 36695182 10.1161/CIR.0000000000001123 PMC12135016

[4] Heidenreich PA Bozkurt B Aguilar D . 2022 AHA/ACC/HFSA guideline for the management of heart failure: a report of the American College of Cardiology/American Heart Association joint committee on clinical practice guidelines. Circulation. 2022;145:e895–1032. 35363499 10.1161/CIR.0000000000001063

[5] Anand IS Gupta P. Anemia and iron deficiency in heart failure: current concepts and emerging therapies. Circulation. 2018;138:80–98. 29967232 10.1161/CIRCULATIONAHA.118.030099

[6] Thompson CB Jakubowski JA Quinn PG Deykin D Valeri CR. Platelet size as a determinant of platelet function. J Lab Clin Med. 1983;101:205–13. 6822760

[7] Levine B Kalman J Mayer L Fillit HM Packer M. Elevated circulating levels of tumor necrosis factor in severe chronic heart failure. N Engl J Med. 1990;323:236–41. 2195340 10.1056/NEJM199007263230405

[8] Rechcinski T Jasinska A Forys J . Prognostic value of platelet indices after acute myocardial infarction treated with primary percutaneous coronary intervention. Cardiol J. 2013;20:491–8. 24469872 10.5603/CJ.2013.0134

[9] Tsutamoto T Hisanaga T Wada A . Interleukin-6 spillover in the peripheral circulation increases with the severity of heart failure, and the high plasma level of interleukin-6 is an important prognostic predictor in patients with congestive heart failure. J Am Coll Cardiol. 1998;31:391–8. 9462584 10.1016/s0735-1097(97)00494-4

[10] Vatankulu MA Sonmez O Ertas G . A new parameter predicting chronic total occlusion of coronary arteries: platelet distribution width. Angiology. 2014;65:60–4. 23636855 10.1177/0003319713486339

[11] Vogiatzis I Samaras A Grigoriadis S Sdogkos E Koutsampasopoulos K Bostanitis I. The mean platelet volume in the prognosis of coronary artery disease severity and risk stratification of acute coronary syndromes. Med Arch. 2019;73:76–80. 31391691 10.5455/medarh.2019.73.76-80 PMC6643353

[12] Zheng YY Wang L Shi Q. Mean platelet volume (MPV) and platelet distribution width (PDW) predict clinical outcome of acute ischemic stroke: a systematic review and meta-analysis. J Clin Neurosci. 2022;101:221–7. 35636058 10.1016/j.jocn.2022.05.019

[13] Sato Y Yoshihisa A Watanabe K . Association between platelet distribution width and prognosis in patients with heart failure. PLoS One. 2020;15:e0244608. 33373413 10.1371/journal.pone.0244608 PMC7771660

[14] Marques I Lopes Ramos R Mendonca D Teixeira L. One-year mortality after hospitalization for acute heart failure: predicting factors (PRECIC study subanalysis). Rev Port Cardiol. 2023;42:505–13. 36893846 10.1016/j.repc.2022.07.017

[15] McMurray JJ Adamopoulos S Anker SD . ESC guidelines for the diagnosis and treatment of acute and chronic heart failure 2012: the task force for the diagnosis and treatment of acute and chronic heart failure 2012 of the European Society of Cardiology. Developed in collaboration with the heart failure association (HFA) of the ESC. Eur J Heart Fail. 2012;14:803–69. 22828712 10.1093/eurjhf/hfs105

[16] Ponikowski P Voors AA Anker SD , ESC Scientific Document Group. 2016 ESC Guidelines for the diagnosis and treatment of acute and chronic heart failure: the Task Force for the diagnosis and treatment of acute and chronic heart failure of the European Society of Cardiology (ESC) developed with the special contribution of the Heart Failure Association (HFA) of the ESC. Eur Heart J. 2016;37:2129–200. 27206819 10.1093/eurheartj/ehw128

[17] Levey AS Coresh J Greene T , Chronic Kidney Disease Epidemiology Collaboration. Using standardized serum creatinine values in the modification of diet in renal disease study equation for estimating glomerular filtration rate. Ann Intern Med. 2006;145:247–54. 16908915 10.7326/0003-4819-145-4-200608150-00004

[18] Nardin M Verdoia M Cao D . Platelets and the atherosclerotic process: an overview of new markers of platelet activation and reactivity, and their implications in primary and secondary prevention. J Clin Med. 2023;12:6074. 37763014 10.3390/jcm12186074 PMC10531614

[19] Scherlinger M Richez C Tsokos GC Boilard E Blanco P. The role of platelets in immune-mediated inflammatory diseases. Nat Rev Immunol. 2023;23:495–510. 36707719 10.1038/s41577-023-00834-4 PMC9882748

[20] Yun SH Sim EH Goh RY Park JI Han JY. Platelet activation: the mechanisms and potential biomarkers. Biomed Res Int. 2016;2016:9060143. 27403440 10.1155/2016/9060143 PMC4925965

[21] Biljak VR Pancirov D Cepelak I Popovic-Grle S Stjepanovic G Grubisic TZ. Platelet count, mean platelet volume and smoking status in stable chronic obstructive pulmonary disease. Platelets. 2011;22:466–70. 21506665 10.3109/09537104.2011.573887

[22] Korniluk A Koper-Lenkiewicz OM Kaminska J Kemona H Dymicka-Piekarska V. Mean platelet volume (MPV): new perspectives for an old marker in the course and prognosis of inflammatory conditions. Mediators Inflamm. 2019;2019:9213074. 31148950 10.1155/2019/9213074 PMC6501263

[23] Vagdatli E Gounari E Lazaridou E Katsibourlia E Tsikopoulou F Labrianou I. Platelet distribution width: a simple, practical and specific marker of activation of coagulation. Hippokratia. 2010;14:28–32. 20411056 PMC2843567

[24] Gladwin AM Martin JF. The control of megakaryocyte ploidy and platelet production: biology and pathology. Int J Cel Cloning. 1990;8:291–8. 10.1002/stem.5530080414 2205666

[25] Kario K Matsuo T Nakao K. Cigarette smoking increases the mean platelet volume in elderly patients with risk factors for atherosclerosis. Clin Lab Haematol. 1992;14:281–7. 1478007 10.1111/j.1365-2257.1992.tb00103.x

[26] Murphy SP Kakkar R McCarthy CP Januzzi JL Jr. Inflammation in heart failure: JACC state-of-the-art review. J Am Coll Cardiol. 2020;75:1324–40. 32192660 10.1016/j.jacc.2020.01.014

[27] Ibrahim NE Januzzi JL Jr. Beyond natriuretic peptides for diagnosis and management of heart failure. Clin Chem. 2017;63:211–22. 28062619 10.1373/clinchem.2016.259564

[28] Wang Z Pan X Xu H . Serum soluble ST2 is a valuable prognostic biomarker in patients with acute heart failure. Front Cardiovasc Med. 2022;9:812654. 35224046 10.3389/fcvm.2022.812654 PMC8863653

[29] George M Jena A Srivatsan V Muthukumar R Dhandapani VE. GDF 15—a novel biomarker in the offing for heart failure. Curr Cardiol Rev. 2016;12:37–46. 26750722 10.2174/1573403X12666160111125304 PMC4807717

[30] Zaborska B Sikora-Frac M Smarz K . The role of galectin-3 in heart failure-the diagnostic, prognostic and therapeutic potential-where do we stand? Int J Mol Sci. 2023;24:13111. 37685918 10.3390/ijms241713111 PMC10488150

